# The TalkMeHome service for people with early dementia

**Published:** 2012-06-15

**Authors:** Marike Hettinga, Jan Nauta, Lammie van den Bosch, Cristian Hesselman, Jeffrey Brangert, Martine de Jong, Marcel Roest, Matti Groot

**Affiliations:** Windesheim University of Applied Sciences, Netherlands; Windesheim University of Applied Sciences, Netherlands; Windesheim University of Applied Sciences, Netherlands; Novay, Netherlands; Carint Reggeland Groep, Netherlands; Carint Reggeland Groep, Netherlands; Verklizan BV, Netherlands; Verklizan BV, Netherlands

**Keywords:** dementia, navigation, remote assistance, independent living

## Abstract

**Introduction:**

People suffering from mild dementia may get lost during a walk, which can be dangerous for them and may add to the anxiety felt by their informal caregivers. TalkMeHome is a new service that allows these people to get home safely in such situations using their mobile phone. With an emergency button they can call a remote care professional who will guide the lost person home. To accomplish this, the professional caregiver is able to follow the person’s GPS-location on a map and in Google Streetview. This paper reports on the gathering of user requirements, the technical design of the TalkMeHome service, and small scale experiments to assess the effectiveness and user experience of the service.

**Aims and objectives:**

The goal of the evaluation of the TalkMeHome service was to assess the effectiveness of this novel service and to investigate how persons with mild dementia and care professionals experience the service. The final aim is to make the TalkMeHome service structurally available for the target group.

**Methods:**

User requirements were collected through desk research, expert interviews with care professionals, and pre-experiments by researchers in which conventional trackers were used combined with an additional mobile phone to simulate the anticipated implementation as close as possible. In the small scale experiments four participants with mild dementia were included using inclusion criteria based on the target group. Each participant was accompanied outdoor by two researchers, one observing the participant, the other ensuring the participant’s safety regarding traffic. The actual test began by pushing the alarm button, simulating being ‘lost’. This established a connection with the care professional who then started guiding the participant back home. Data were collected by observation, interviews with the participants, and questionnaires and interviews with the care professionals.

**Results:**

As for effectiveness, all four participants were guided home satisfactorily, technical imperfections set aside. Only a few easily corrected mistakes were noticed. The evaluations further showed that guiding people with dementia home is possible even under suboptimal conditions (unreliable information of the person with dementia, missing location updates or an incomplete map). Regarding the experiences of the users, both persons with dementia and care professionals, we conclude that the contact between both was easy but guiding somebody home is a demanding task for a care professional.

**Conclusions:**

The results suggest that TalkMeHome is an effective service, which increases our confidence that it can be made available as a commercial service. It also shows that a technology that is not 100% accurate can still provide an added value. In terms of user experience, our work revealed the difficulty of the care professional’s task. With an aging population the demand for appropriate support will increase. For this reason the research group ‘ICT-Innovations in Healthcare’ will take the initiative for a ‘Skills Lab’, a laboratory equipped with the necessary hardware and software to facilitate both research and training with regard to the skills of a care professional offering remote services to patients.

